# Programmed cell death and *Salmonella* pathogenesis: an interactive overview

**DOI:** 10.3389/fmicb.2023.1333500

**Published:** 2024-01-04

**Authors:** Yu Zhang, Maodou Xu, Yujiao Guo, Li Chen, Wanwipa Vongsangnak, Qi Xu, Lizhi Lu

**Affiliations:** ^1^State Key Laboratory for Managing Biotic and Chemical Threats to the Quality and Safety of Agro-Products, Zhejiang Academy of Agricultural Sciences, Hangzhou, Zhejiang, China; ^2^College of Animal Science and Technology, Yangzhou University, Yangzhou, Jiangsu, China; ^3^Department of Zoology, Faculty of Science, Kasetsart University, Bangkok, Thailand

**Keywords:** programmed cell death, salmonella, apoptosis, pyroptosis, necroptosis, autophagy

## Abstract

Programmed cell death (PCD) is the collective term for the intrinsically regulated death of cells. Various types of cell death are triggered by their own programmed regulation during the growth and development of organisms, as well as in response to environmental and disease stresses. PCD encompasses apoptosis, pyroptosis, necroptosis, autophagy, and other forms. PCD plays a crucial role not only in the growth and development of organisms but also in serving as a component of the host innate immune defense and as a bacterial virulence strategy employed by pathogens during invasion. The zoonotic pathogen *Salmonella* has the ability to modulate multiple forms of PCD, including apoptosis, pyroptosis, necroptosis, and autophagy, within the host organism. This modulation subsequently impacts the bacterial infection process. This review aims to consolidate recent findings regarding the mechanisms by which *Salmonella* initiates and controls cell death signaling, the ways in which various forms of cell death can impede or restrict bacterial proliferation, and the interplay between cell death and innate immune pathways that can counteract *Salmonella*-induced suppression of host cell death. Ultimately, these insights may contribute novel perspectives for the diagnosis and treatment of clinical *Salmonella*-related diseases.

## 1 Introduction

Programmed cell death (PCD) is a pervasive phenomenon in the growth and development of organisms, characterized by a genetically regulated process of active and organized cell death. Specifically, PCD involves the initiation of a protective mechanism in response to cytokine stimulation from either the internal or external environment. This mechanism entails the activation of gene coding and protein metabolism. Initially, apoptosis was acknowledged as the prevailing form of cell death in the early stages of PCD research, while necrosis was predominantly viewed as an incidental consequence of external stimuli leading to cell death. Necrosis is commonly defined as the uncontrolled death of a cell, typically occurring after a significant injury, leading to the release of cellular contents into neighboring tissues and subsequent harm (D'Arcy, 2019). As investigations into this genetically regulated, self-contained, and organized form of cell death progress, an increasing number of cell death modalities have been unveiled. In the context of pathogenic bacterial infections, these cell deaths are commonly classified into various prevalent forms of PCD, such as apoptosis, pyroptosis, necroptosis, autophagy, and ferroptosis, based on discernible morphological alterations, essential biochemical factors, immune pathway components, and effector proteins (Table 1).

**Table 1:** Comparison of apoptosis, pyroptosis, necroptosis, and autophagy.

**PCD form**	**Morphological characteristics**	**Biochemical characteristics**	**Immunological characteristics**	**Key effector proteins**
Apoptosis	Reduced cell and nuclear volume, chromatin condensation, nuclear fragmentation, apoptotic vesicle formation, and cytoskeletal disintegration	Caspase activation, DNA degradation fragmentation	Usually does not elicit an inflammatory response, anti-inflammatory, and in some cases elicits an immune response through exposure and release of DAMPs	Caspase, Bcl-2, Bax, P53, Fas
Pyroptosis	Plasma membrane rupture, release of cell contents and pro-inflammatory cytokines	Caspase1 and Caspase11 activation, generation of active IL-1β, and IL-I8, GSDMD protein hydrolysis activation	Release of DAMPs, pro-inflammatory	GSDM protein family, Caspase-1/3/4/5/8/11, Inflammasomes
Necroptosis	Swelling of cells and organelles, moderate condensation of chromatin, rupture of cell membranes, and spillage of cellular components	Decreased levels of ATP. RIP1, RIP3, and MLKL activation	Usually releases DAMPs pro-inflammatory and in some cases anti-inflammatory	RIP1 and RIP3
Autophagy	Massive autophagic vesicle formation	LC3-I to LC3-I1, Self-substrate (e.g., p62) degradation	Usually inhibits the activation of inflammasomes to act as an anti-inflammatory, and in some cases pro-inflammatory	ATG5, ATG7, LC3, Beclin-1, DRAM3, and TFEB
Ferroptosis	Reduced mitochondrial volume, increased density of bilayer membranes, reduction or disappearance of mitochondrial cristae, and rupture of the outer mitochondrial membrane	Iron accumulation and lipid peroxidation	Release of DAMPs and pro-inflammatory	GPX4, TFR1, ferritin, SLC7A11, NRF2, P53, ACSL4, and FSP1

Host cell death is a prevalent characteristic observed in numerous bacterial infections, serving as a means to restrict bacterial replication. Conversely, bacteria employ a diverse range of virulence factors or effector proteins to counteract or manipulate the signaling pathways associated with cell death, thereby evading or impeding cell death induction. For instance, the T3SS effectors of *Salmonella* Typhimurium have been identified to interact with apoptotic, necroptotic, and pyroptotic cell death cascades, thereby impeding the efficient elimination of the bacteria and the recruitment of neutrophils or dendritic cells to the site of infection (LaRock et al., 2015). PCD is widely recognized as an integral component of the innate immune defense in host organisms. *Salmonella*, a zoonotic pathogen, is a facultative intracellular pathogen that poses a significant threat to both global human and animal health, resulting in substantial economic burdens. This pathogen consistently influences the progression and resolution of infections by stimulating the production of cytokines with potent immunomodulatory properties and the secretion of various virulence effector proteins via the bacterial type III secretion system (T3SS), which governs the regulation of PCD (Wemyss and Pearson, 2019). However, the type of cell death induced during a bacterial infection is contingent upon various factors, including the infecting pathogen, its virulence factors, the specific tissues and cell types affected, and the host's inflammatory response. This review aims to provide a comprehensive overview of the molecular mechanisms governing PCD in *Salmonella* infections, with a particular emphasis on apoptosis, pyroptosis, necroptosis, and autophagy, as well as their interplay in the containment or eradication of *Salmonella* during infections. The insights presented herein offer novel targets and ideas for the treatment of diseases associated with *Salmonella* infection.

## 2 Apoptosis induced by activation of caspases by *Salmonella* infection

**Figure 1:**
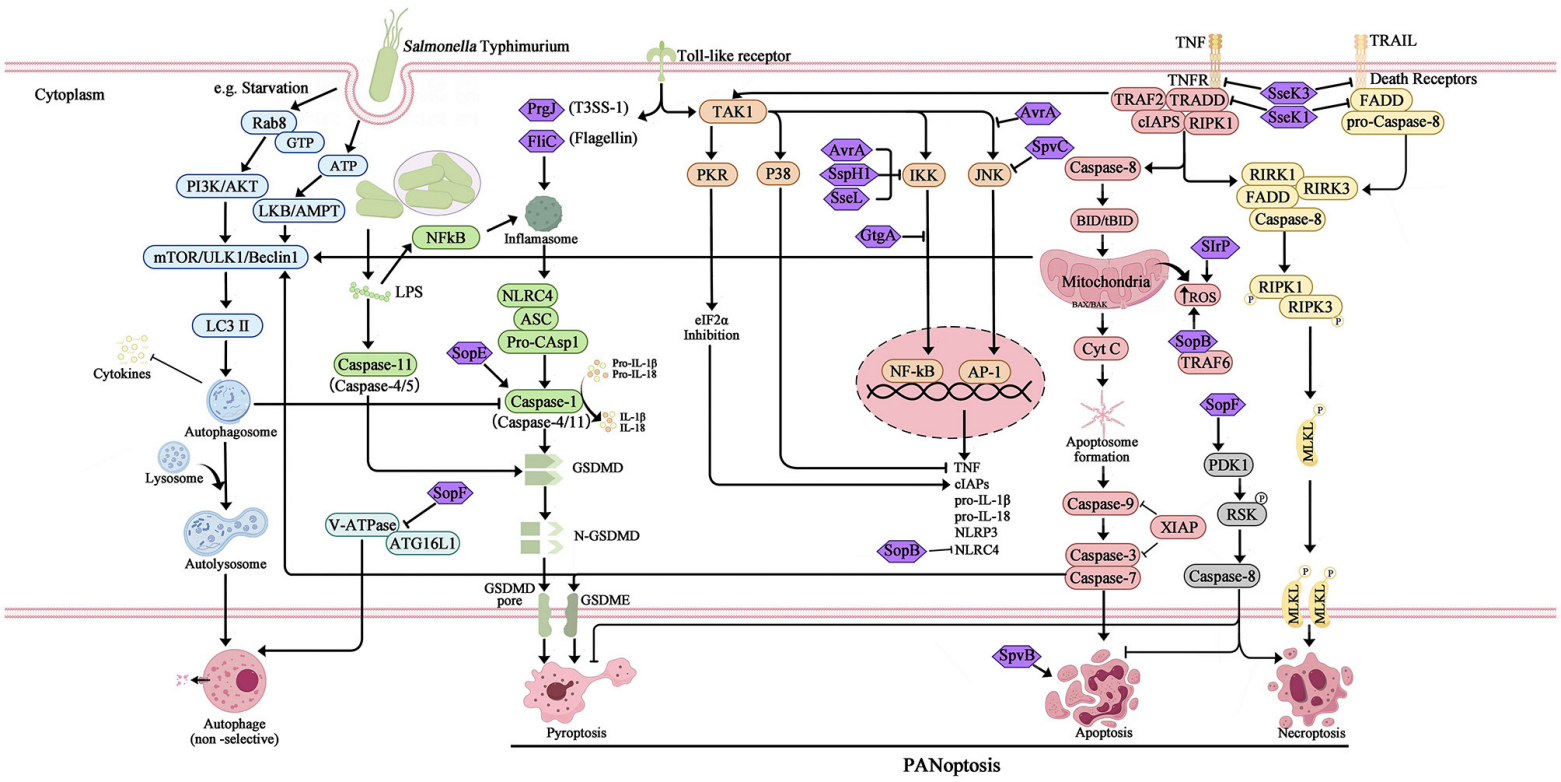
Overview of the different type of programmed cell death and the interference of T3SS effector proteins during *Salmonella* Typhimurium infection. The activation and inhibition of apoptosis, pyroptosis, necroptosis, and canonical autophagy are mediated by the SPI-1 and SPI-2 T3SS effector proteins, which interact with host innate immune pathways during *Salmonella* Typhimurium infection. The extensive overlap between apoptosis, pyroptosis, and necroptosis results in a phenomenon known as PANoptosis was also induced during *Salmonella* Typhimurium infection.

Apoptosis is a regulated process of cellular death, wherein the activation of Caspases serves as the principal mechanism (Stringer et al., 2023). It is triggered in cells that have been subjected to either internal or external signaling cascades due to damage or stress. The initiation of apoptosis is contingent upon the activation of apoptotic Caspases and is marked by various cellular changes, including the formation of apoptotic vesicles, cell membrane blebbing, cell shrinkage, DNA fragmentation, and nucleoplasmic condensation (Guiney, 2005). The pathways of Caspase dependency encompass both the extrinsic death pathway mediated by death receptors and the intrinsic death pathway mediated by mitochondria. The extrinsic death pathway is triggered when ligands or cytokines bind to transmembrane death receptors located on the cell surface, thereby regulating apoptosis through the activation of the death-inducing signaling complex (DISC) and Caspases-8. Specifically, the death receptor Fas binds to the ligand FasL and interacts with the Death domain (DD) on the protein, leading to the recruitment of the Fas-associated death domain protein (FADD). FADD possesses a Death effector domain (DED) that facilitates the interaction between FADD and other DED-containing proteins, such as Caspase-8. FADD undergoes aggregation with Caspase-8 to form the DISC. Upon activation, Caspase-8 undergoes a conformational change from a single-stranded zymogen to a biologically active double-stranded protein. This activated Caspase-8 then further activates Caspase-3, initiating the extrinsic death pathway (Grassme et al., 2001). On the other hand, the intrinsic death pathway is triggered by the release of apoptotic proteins due to internal stimulation. Various factors such as microbial infection, DNA damage, cytotoxic stimulation, and other influences lead to the oligomerization of B cell lymphoma 2 (Bcl-2) family proteins Bax and Bak (Stringer et al., 2023). This oligomerization increases the permeability of the mitochondrial membrane, resulting in the formation of annular lipid pores in the intermembrane space of the mitochondria. Cytochrome C (CytC) and other pro-apoptotic factors are released into the cytoplasm via perforated mitochondria. In the presence of dATP, CytC and apoptotic protease-activating factor 1 (Apaf-1) with CARD domain bind to Caspase-9, forming an apoptotic complex. This complex can activate Caspase-9, which in turn triggers the hydrolysis and processing activation of apoptosis execution proteins Caspase-3 and Caspase-7 into their mature forms. Ultimately, this cascade leads to intrinsic cell death (Ketelut-Carneiro and Fitzgerald, 2022) (Figure 1).

**Figure 2:**
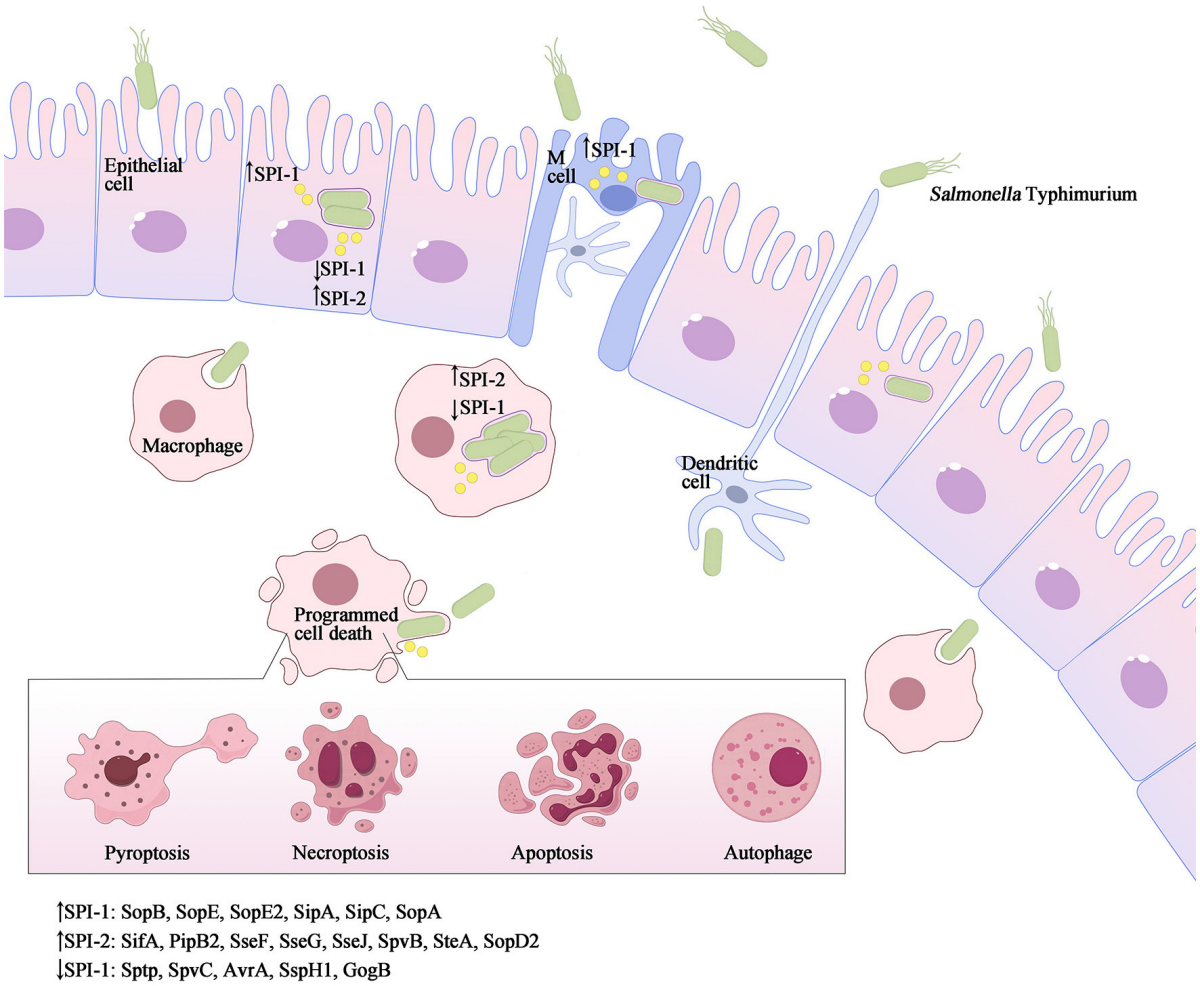
*Salmonella* Typhimurium invasion elicits various forms of programmed cell death. As a facultative intracellular pathogen, *Salmonella* Typhimurium can infect a range of cell types such as macrophages, dendritic cells, and epithelial cells. *Salmonella* Typhimurium accomplishes intestinal epithelium traversal by invading M-cells located above Peyer's patches, and it can also be captured by phagocytic cells such as dendritic cells or macrophages from the intestinal lumen. Once internalized, the SPI-1 T3SS and its effectors are downregulated, while SPI-2 is upregulated to promote the formation of *Salmonella*-containing vacuoles (SCVs) and facilitate *Salmonella* replication. Throughout the infection process, both SPI-1 and SPI-2 effector proteins interact with host innate immune pathways, either activating or inhibiting inflammatory responses and inducing different types of programmed cell death, such as apoptosis, pyroptosis, necroptosis, autophagy and others.

The foodborne pathogen *Salmonella* is acquired through the ingestion of contaminated food and subsequently enters the digestive tract. During the initial phase of *Salmonella* infection, the pathogen infiltrates intestinal epithelial cells by employing SPI-1 effectors, which induce alterations in intestinal folds and actin structures. This process facilitates the uptake of *Salmonella* by non-phagocytic cells. Alternatively, *Salmonella* may traverse M cells that cover the Peyer's patches, allowing entry into the lymphoid follicles and lamina propria, where they are captured by phagocytes such as dendritic cells and macrophages. Upon internalization by host cells and residing within specialized membrane-bound blebs known as *Salmonella*-containing vacuoles (SCVs), the expression of SPI-1 T3SS and its associated effectors is down-regulated, while SPI-2 is up-regulated (LaRock et al., 2015; Wemyss and Pearson, 2019). This regulatory shift promotes the formation of SCVs and facilitates the replication of *Salmonella* (Figure 2). Within these vacuoles, the bacteria employ the T3SS to deliver various effector proteins into the cytoplasm of phagocytes, thereby triggering Caspase-1 activation and subsequent cell death. The release of SipB leads to rapid cell death, exhibiting characteristics of both necrosis and apoptosis, along with the release of the proinflammatory cytokines IL-1β and IL-18 (Boise and Collins, 2001). The second mechanism primarily involves the activation of Toll-like receptor 4 (TLR4) by *Salmonella* through various components such as lipopolysaccharide (LPS) in the cell wall, intracellular TLR4 adapter proteins, and kinase pathways. This activation initiates a complex cascade of signal transduction, leading to the induction of both pro- and anti-apoptotic factors. Specifically, IKK and JNK activate the transcription factors NF-κB and AP-1, which are pivotal in the stimulation of pro-inflammatory cytokine genes, including TNF-α. Additionally, p38-MAPK and AKT play crucial roles in the induction of anti-apoptotic proteins (Guiney, 2005). On the other hand, the kinase PKR plays a role in facilitating *Salmonella*-induced apoptosis through its ability to impede protein synthesis via eIF2a phosphorylation. Additionally, PKR and type I interferons stimulate the activation of interferon response factor 3 (IRF3), which further triggers the production of pro-apoptotic factors within phagocytes (Hsu et al., 2004). The equilibrium between these apoptotic regulators ultimately dictates the fate of a cell, determining whether it will endure or undergo PCD. Notably, Caspase-3 assumes a pivotal role as the principal executor of these distinctive apoptotic alterations. However, *Salmonella* is known to produce several effector proteins that have the potential to influence the apoptotic pathway. Specifically, the SPI-1 T3SS facilitates the secretion of SptP, AvrA, and SspH1 (also secreted by SPI-2), which in turn promote apoptosis by inhibiting NF-κB, down-regulating MAPK signaling, or facilitating the secretion of SigD (SopB) to activate the AKT effect and delay apoptosis. Additionally, the SPI-2 T3SS mediates the secretion of SpvB, which leads to the depolymerization of the actin cytoskeleton in macrophages, thereby contributing to SPI-2-dependent apoptosis (Schleker et al., 2012; Wemyss and Pearson, 2019) (Figure 1).

## 3 *Salmonella* induces inflammasome-mediated pyroptosis

**Figure 3:**
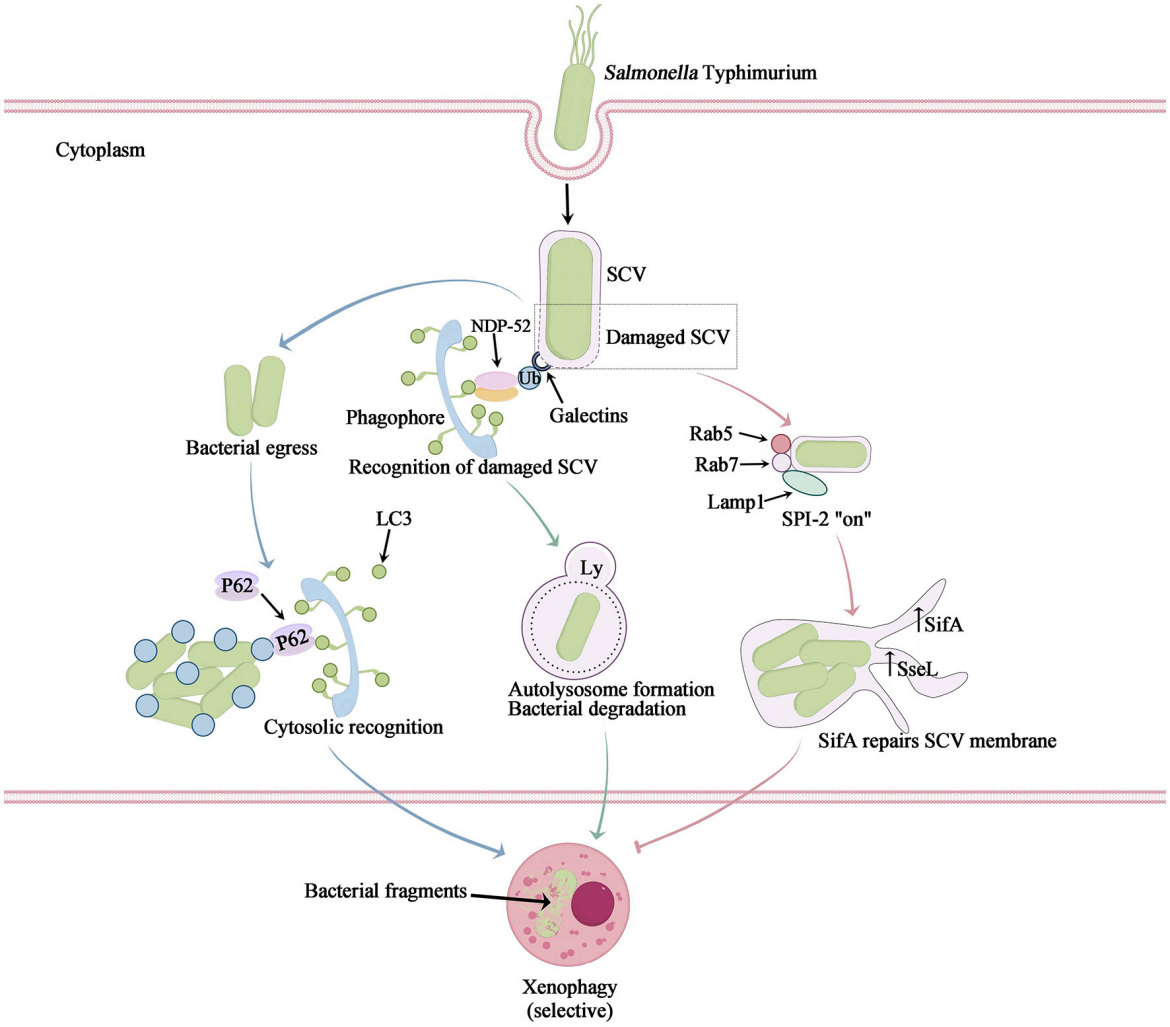
The mechanism of intracellular *Salmonella* Typhimurium-induced xenophagy. *Salmonella* Typhimurium infection not only causes cell nutrient deprivation or stimulation leading to non-selective autophagy, but also triggers selective autophagy to clear bacteria, also known as xenophagy. After their escape from a *Salmonella*-containing vacuole (SCV), *Salmonella* bacteria are marked with ubiquitin. Autophagy adaptors then bind to the ubiquitylated bacteria and link them to LC3-II on the initial autophagosome membrane (blue arrow). The SCV is damaged by T3SS-1, leading to the recruitment of galectin-8 to bind ubiquitin and the recruitment of NDP52, which connects LC3 and promotes autophagosome maturation. This process exposes internal proteins of the SCV membrane to the cytosol and causes sharp changes in ion concentration, potentially resulting in the formation of an ubiquitinated SCV membrane. The autophagic machinery recognizes this ubiquitinated membrane (green arrow). Autophagy facilitates the restoration of the SCV membrane damaged by T3SS-1 and subsequently induces acidification to generate SIFs, thereby enabling bacterial survival and replication within (pink arrow).

Pyroptotic cell death is characterized by the progressive enlargement of cells until the rupture of the plasma membrane, leading to the formation of a pore. This pore allows for the release of intracellular contents, including the cytokines IL-1β and IL-18, resulting in a robust inflammatory response (Ketelut-Carneiro and Fitzgerald, 2022). The classical pathway of pyroptosis is regulated by Caspase-1, which undergoes processing and activation within the inflammasomes. These inflammasomes are supramolecular complexes that consist of NLRs, typically featuring C-terminal leucine-rich repeat sequences (LRRs), and are responsible for sensing signals from bacteria (Frank and Vince, 2019). Furthermore, nucleotide-binding domain and leucine-rich repeat containing receptors (NLRs) possess a variable N-terminal region housing the pyrin structural domain (PYD). Upon stimulation of the host by signals emanating from bacterial infection, inducible pattern recognition receptors (PRRs) engage with pathogen-associated molecular patterns (PAMPs) and assemble an inflammasome alongside the pyroptosis adapter protein ASC. This assembly catalyzes the activation of Caspase-1, which subsequently cleaves the precursors of IL-1β and IL-18, resulting in the formation of biologically active inflammatory cytokines (Fink and Cookson, 2007). IL-1β is an endogenous pyrogen that elicits fever, stimulates the expression of cytokines and chemokines, and facilitates the migration of leukocytes. Additionally, IL-18 triggers the production of IFN-γ, which plays a crucial role in the activation of T cells and macrophages (Guiney, 2005). Furthermore, the activation of the inflammasome-associated Caspase-1 can cleave Gasdermin D (GSDMD), leading to the formation of structural domain proteins containing the active fragment of GSDMD-N (Fink and Cookson, 2007). This active fragment induces the perforation and rupture of the cell membrane, resulting in the rapid release of IL-1β and IL-18 into the extracellular environment, thereby instigating inflammatory responses and pyroptosis (Brokatzky and Mostowy, 2022) (Figure 3).

Pyroptosis serves as a vital innate immune response against *Salmonella*, influencing the progression of bacterial infection by inducing pyroptosis in both immune and non-immune cells. The activation of distinct inflammasomes in *Salmonella*-induced pyroptosis is contingent upon the virulence of the *Salmonella* strain and the response of the infected cell. During the *Salmonella* Typhimurium infection, the activation of host cell NLR family apoptosis inhibitory proteins (NAIP) occurs upon sensing of flagellin (FliC and FljB) or PrgJ (an SPI-1 secreted protein). These proteins subsequently interact with protein 4 (NLRC4), which possesses the NLR family Caspase recruitment domain (CARD), resulting in the formation of a complex with ASC. This complex then catalyzes the hydrolytic activation of Caspase-1, thereby mediating cellular pyroptosis (Fink and Cookson, 2007; Wemyss and Pearson, 2019). Additionally, NLRP3 (which detects increased ROS or K^+^ efflux) and pyrin (which senses inhibition of RhoA GTPase activity) can also induce pyroptosis through ASC Caspase-1 inflammasomes (Wemyss and Pearson, 2019). Both NLRC4 and NLRP3 are involved in the maturation of IL-1β and IL-18, as well as the induction of pyroptosis, in macrophages infected with *Salmonella* Typhimurium (Vladimer et al., 2013). In the non-classical pathway of pyroptosis, the LPS from *Salmonella* directly interacts with Caspase-4/5/11 in macrophages, leading to the activation of Caspase-4/5/11 (Knodler et al., 2014). This activated Caspase then cleaves GSDMD protein, triggering the activation of NLRP3 inflammasomes and subsequent activation of Caspase-1. This cascade ultimately results in the release of IL-1β, IL-18, and the occurrence of pyroptosis (Ma et al., 2021). Additionally, NLRC4 and the activation of non-classical inflammasomes are implicated in the response of epithelial cells to *Salmonella* infection, potentially contributing to the containment of bacterial dissemination within the intestinal mucosa.

*Salmonella* is known to generate various effector proteins that have the potential to impact the pyroptosis pathway. In the case of *Salmonella* Typhimurium-infected HeLa and RAW264.7 cells, as well as *in vivo* infection of mouse enterocytes, the *Salmonella* Typhimurium type III effector protein (SopE) was observed to stimulate the activation of host cell Rac1, which is a subunit of the Rho GTPase. This activation subsequently induces Caspase-1 activation and the secretion of IL-1β (Müller et al., 2009; Hoffmann et al., 2010). SopB has been found to be linked with the downregulation of NLRC4 in *Salmonella* Typhimurium-infected macrophages and B cells (Hu et al., 2017). The absence of NLRC4, a crucial inflammasome, hinders the pyroptosis response to *Salmonella* Typhimurium infection, thereby providing bacteria with an enhanced opportunity for replication prior to evading the host cell (García-Gil et al., 2018). SipB which interacts with SipC to form a translocon pore, facilitating SPI-1 effector translocation into the host cell is reportedly sufficient to induce Caspase-1-mediated “apoptosis” and IL-18 maturation in SipB transfected or *Salmonella* Typhimurium-infected (Hersh et al., 1999; van der Velden et al., 2003). Overall, *Salmonella* infection process is capable of activating different inflammasomes, causing cellular focalization and enhancing bacterial invasion and immune escape from the host cell.

## 4 *Salmonella* infection induces necroptosis dependent on the classical pathway TNF signaling

Necrosis is characterized as a passive and unregulated form of cell death, whereas necroptosis adheres to intracellular signaling regulation, exhibiting Caspase activity-independent characteristics. Necroptosis is visually manifested through cell swelling, mitochondrial dysfunction, plasma rupture, and subsequent release of intracellular contents, which incites an inflammatory response in the surrounding tissue (Blériot and Lecuit, 2016). The canonical pathway of necroptosis, known as the tumor necrosis factor (TNF) signaling pathway, necessitates the participation of various kinases, including receptor-interacting serine/threonine protein kinase 1/3 (RIPK1/RIPK3)and mixed-lineage kinase domain-like protein (MLKL). Furthermore, the process of necroptosis necessitates the involvement of specialized receptors and ligands located on the plasma membrane, primarily mediated by tumor necrosis factor-α (TNF-α) and tumor necrosis factor receptor 1 (TNFR1). Upon ligation of TNF-α and TNFR1, RIPK1 and TNFR1-associated death domain protein (TRADD) are recruited, leading to the formation of a multimeric complex 1. This multimeric complex I comprises not only RIPK1 and TRADD, but also cellular inhibitors of apoptosis 1/2 (cIAP1/2), tumor necrosis factor receptor-associated factor 2/5 (TRAF2/5). Additionally, cIAP1/2 is capable of inducing the ubiquitination of RIPK1 and facilitating the up-regulation of anti-apoptotic genes. When the deubiquitinase CYLD deubiquitinates RIPK1, it results in a reduction in the stability of multimeric complex I. This reduction leads to the formation of a multimeric complex consisting of RIPK1, FADD, TRADD, RIPK3, and Caspase-8. The activation of multimeric complex II triggers the downstream signaling pathway of apoptosis. Caspase-8 then cleaves and inactivates RIPK1 and RIPK3, initiating the Caspase cascade reaction, ultimately resulting in cell apoptosis (Hu and Zhao, 2013; Wemyss and Pearson, 2019). However, bacterial pathogens hinder the activity of Caspase-8, preventing the cleavage and inactivation of RIPK1 and RIPK3 by Caspase-8. RIPK1 and RIPK3 form a complex and initiate phosphorylation, leading to the recruitment of the downstream necrosis execution protein MLKL and the subsequent formation of necrosomes (Hu and Zhao, 2013). Within these bodies, RIPK3 phosphorylates MLKL, resulting in the oligomerization and translocation of phosphorylated MLKL to the plasma membrane. At the plasma membrane, phosphorylated MLKL interacts with phosphatidylinositol phosphate (PIP), thereby increasing the permeability of the cytoplasmic membrane and facilitating the release of highly inflammatory damage-associated molecular patterns (DAMPs) into the extracellular space (Frank and Vince, 2019). This process ultimately leads to cell rupture and the induction of cell necroptosis (Figure 1).

During *Salmonella* Typhimurium infection, virulence effector proteins are involved in facilitating the host necroptotic response. Specifically, *Salmonella* secretes effector proteins SseK1 and SseK3, which glycosylate TNFR superfamily members and TRADD or FADD. This glycosylation process inhibits TNF-mediated NF-κB signaling and prevents cell death through apoptosis or necroptosis. Mass spectrometry analysis has identified TNFR1 and TRAIL-R as the glycosylation targets of SseK3, while TRADD is the preferred binding target of SseK1 (Günster et al., 2017; Xue et al., 2020). In addition, SopF was found could activate phosphoinositide-dependent protein kinase-1 (PDK1) to phosphorylate p90 ribosomal S6 kinase (RSK) which down-regulated Caspase-8 activation, resulting in inhibition of pyroptosis and apoptosis, but promotion of necroptosis (Yuan et al., 2023).

## 5 Bidirectional regulation of autophagy induced by *Salmonella* infection

Both apoptosis and autophagy are activated in response to metabolic stress. Growth factor deprivation, limitation of nutrients and energy metabolism, activate the mTOR/ULK1/Beclin1 pathway, the multiple effects of some key proteins such as Caspase-3, Beclin1, and p53 lead to the complex interplay between these two pathways (Nikoletopoulou et al., 2013). However, *Salmonella* infection not only causes cell nutrient deprivation or stimulation leading to non-selective autophagy, but also triggers selective autophagy to clear bacteria, also known as xenophagy. *Salmonella* infection elicits non-selective autophagy, primarily triggered by amino acid deficiency, resulting in the suppression of the cell growth regulator mTOR. The PI3K-Akt-mTOR signaling pathway plays a pivotal role in this process (Miller et al., 2020) (Figure 1). Furthermore, xenophagy which is the activation of selective autophagy, is widely recognized as a promising strategy to combat bacterial infections. Xenophagy exerts its influence on *Salmonella* infection through three distinct pathways, two of which function as antibacterial defense mechanisms, effectively targeting invading *Salmonella*, eradicating pathogens, and subsequently safeguarding host cells. Additionally, *Salmonella*-infected cells employ an alternative pathway that not only facilitates the restoration of the damaged SCV, but also fosters an environment conducive to the replication and survival of the pathogen itself (Owen and Casanova, 2015; Zheng et al., 2022) (Figure 3). In the initial route, a portion of *Salmonella* bacteria possess the ability to evade this vacuole. Once these bacteria infiltrate the cytoplasm, they become tagged with unbound ubiquitin and undergo selective autophagy. Adapter proteins, namely SLRs (such as SQSTM1/p62, NDP52, and OPTN proteins), are recognized for their capacity to bind to ubiquitin-labeled bacteria. Additionally, these adapter proteins function as autophagy substrates, binding to LC3 in order to facilitate the mediation of autophagosomes. Specifically, it accomplishes this by means of the ubiquitin-binding domain and ubiquitin-labeled bacteria. It establishes a bond with characterized bacteria and engages with the autophagosome molecular marker Atg8/LC3 as well as the autophagy substrate SQSTM1/p62, thereby facilitating its association with the developing autophagosome. Consequently, it encapsulates *Salmonella* within the autophagosome and subsequently breaks down the bacteria. Another mechanism by which cells defend against *Salmonella* autophagy involves the recognition of damage to the SCV membrane caused by the pinhole apparatus of SPI-1 T3SS (Mostowy, 2013). In this scenario, *Salmonella* is found within the damaged SCV. The perforation of the SCV membrane, induced by SPI-1 T3SS, attracts various cytosolic components, including galectin-8. Galectin-8 serves as a marker protein for intact membrane structure and also acts as an inducer of autophagy. The galectin-8-labeled SCV then recruits LC3 and NDP52 to the cell membrane, thereby promoting the generation of autophagy signals. Consequently, this process leads to the autophagic capture and degradation of *Salmonella* (Wu et al., 2020).

*Salmonella*, being a parthenogenetic intracellular bacterium, has the ability to enhance its own survival through the induction of cellular autophagy. Unlike autophagy, which serves as a natural cellular immune defense mechanism, *Salmonella* can secrete certain effector proteins to evade fusion with lysosomes during autophagy, allowing for persistent proliferation within the SCV (Wang et al., 2018). For instance, *Salmonella* Typhimurium possesses a virulence plasmid that encompasses a conserved region of approximately 8 kb, which plays a crucial role in bacterial serum resistance, adhesion, and colonization. The spv region of the *Salmonella* plasmid virulence gene encompasses three genes associated with virulence, namely the transcriptional regulator spvR, as well as the structural genes spvB and spvC. Among these, spvB plays a crucial role in determining the extent of *Salmonella* proliferation within macrophages, while also inducing late apoptosis in host cells during *Salmonella* infection. The SPV proteins are essential for ADP-ribosyltransferase activity, which serves as an endotoxin and covalently modifies monomeric actin, thereby creating a favorable growth environment for *Salmonella* infection (Shintani and Klionsky, 2004; Xie et al., 2020). The upregulation of the autophagosome molecular marker Atg8/LC3 facilitates the repair and maturation of the SCV membrane. This process is accompanied by the recruitment of essential membrane-loading molecules Rab5 and Rab7, which ensure cellular maturation and enable the expression of the SPI-2 T3SS. Consequently, the effector protein filamentous body SifA is produced to maintain the integrity of the SCV membrane, further promoting SCV maturation and facilitating the replication of *Salmonella* within the host cell, thereby enhancing its survival (Birmingham and Brumell, 2006). During this temporal phase, the SPI-1 T3SS-mediated damage to the SCV leads to the liberation of ubiquitin and galectin-8 signaling molecules, thereby facilitating the internalization of crucial antimicrobial autophagic constituents (Owen and Casanova, 2015; Xie et al., 2020). Concurrently, the SPI-2 T3SS effector protein, SseL, is activated, culminating in the fulfillment of intracellular *Salmonella* replication through the reduction of autophagic fluxes. Consequently, this reduction instigates apoptosis in macrophages (Mesquita et al., 2012) (Figure 3).

In the initial stages of autophagic activation, the bacteria secrete effector proteins to impede the recognition of PAMPs, thereby evading cellular autophagy (Wang et al., 2018). During the process of autophagy, *Salmonella* fluid releases multiple effector proteins through either simulation or direct covalent modification of host proteins, thus facilitating their escape mechanisms (Table 2). Furthermore, *Salmonella* Typhimurium counteracts host SLRs and releases the effector protein SopA, which promotes the ubiquitination and subsequent degradation of TRIM56 and TRIM65, thereby suppressing IFN-γ-driven autophagy (Zhang et al., 2006). *Salmonella* Typhimurium has developed the membrane proteinase IcsP as a means of evading recognition by the C3 complement system and escaping cell-autonomous autophagy (Sorbara et al., 2018). The effector protein SrrB plays a role in disrupting the activation of the AMPK pathway, leading to the degradation of the AMPK/Sirt1/LBK1 complex. This disruption by SrrB results in an increase in mTOR levels and the inhibition of ULK complex formation (Ganesan et al., 2017). Recent studies have revealed that *Salmonella* effector proteins SseF and SseG disrupt the interaction between the guanine nucleotide exchange factor of GTPase Rab1A and transporter protein particle III, impede the assembly of the ULK1 complex, and reduce the synthesis of PI3P. These actions ultimately inhibit the nucleation of autophagosomes and membrane elongation. Additionally, it has been discovered that the *Salmonella* effector protein SopF can specifically modify V-ATPase, a crucial protein involved in sensing bacterial infection and recruiting autophagy protein ATG16L1 to initiate xenophagy. This alteration inhibits the V-ATPase-ATG16L1 pathway, thereby promoting the proliferation and spread of *Salmonella* within the host (Xu et al., 2019, 2022). In a typical physiological state, mannose-6-phosphate receptors (M6PR) facilitate the transportation of synthetic hydrolases from the trans-Golgi network (TGN) to endocytic lysosomes. However, the *Salmonella*-secreted effector SifA, in conjunction with the small guanosine triphosphatase Rab9, obstructs M6PR transport and reduces hydrolase activity (McGourty et al., 2012). Consequently, this enhances bacterial survival within the host cell.

**Table 2:** *Salmonella* effectors intervention in programmed cell death.

**PCD form**	**Effector protein**	**T3SS**	**Cells affected**	**Mechanism**	**References**
Apoptosis	SlrP	1/2	HeLa	SPI-1 and SPI-2 translocated E3 ubiquitin ligases that interact with thioredoxin-1 (Trx1) and ER chaperone protein ERdj3	Bernal-Bayard et al., 2010; Zouhir et al., 2014
Apoptosis	SpvB	2	HMDM CHO HT-29	Making ADP-ribosylated actin and disrupting the cytoskeleton of eukaryotic cells	Lesnick et al., 2001; Browne et al., 2002; Paesold et al., 2002
Apoptosis	SopB (SigD)	1	MEFs Henle407	Inhibits production of mitochondrial superoxide ROS Activates MAPK and NF-κB signaling through stimulation of Rho-family GTPases	Ruan et al., 2014, 2016
Apoptosis	AvrA	1	HeLa HCT116 HEK293T	Inhibits NF-κB and JNK pro-inflammatory pathways	Collier-Hyams et al., 2002; Ye et al., 2007; Jones et al., 2008
Apoptosis	SseK3	2	HeLa, HEK293	SseK3 Binds TRIM32 and modulates the host's NF-κB signaling activity	Yang et al., 2015
Necroptosis	SseK1	1/2	RAW264.7	Arginine glycosylation of FADD inhibits TNF-induced NF-κB signaling	Günster et al., 2017
Necroptosis	SseK3	2	RAW264.7	Arginine glycosylation of TRADD inhibits TNF-induced NF-κB signaling	Günster et al., 2017
Necroptosis	SopF	1	IECs	PDK1 to phosphorylate RSK which down-regulated Caspase-8 activation	Yuan et al., 2023
Pyroptosis	PrgJ	1	BMMs	Activates caspase-1 through NLRC4	Rayamajhi et al., 2013
Pyroptosis	SlrP	1/2	IECs	Inhibition of IL-1β activation	Rao et al., 2017
Pyroptosis	SipB	1	RAW264.7 DC	SipB induces macrophage apoptosis by binding to caspase-1 SipB induces IL-18 activation and release in human dendritic cells	Hersh et al., 1999; van der Velden et al., 2003
Pyroptosis	SopE	1	HeLa RAW264.7	SopE-driven Rho GTPase-mediated caspase-1 activation	Müller et al., 2009; Hoffmann et al., 2010
Pyroptosis	SopB (SigD)	1	BMDM B Cells	SopB inhibits IL-1β secretion and caspase-1 activation. SopB triggers the PI3K-Akt-YAP pathway to inhibit the NLRC4 inflammasomes	Hu et al., 2017; García-Gil et al., 2018
Autophagy	SopB (SigD)	1	B Cells	SopB activates mTORC1 and inhibits autophagy by phosphorylating ULK1 at its Ser757	Luis et al., 2022
Autophagy	SopF	1	HeLa	SopF specifically modifies V-ATPase to inhibit the autophagic recognition	Xu et al., 2022
Autophagy	SseL	2	HeLa	SseL inhibits selective autophagy of cytosolic aggregates	Mesquita et al., 2012
Autophagy	SopA	1	HeLa HEK293T	Interacting with Human RMA1 Promote the escape of *Salmonella* from the SCVs Stimulates inflammation targeting TRIM56 and TRIM65	Zhang et al., 2006; Kamanova et al., 2016
Autophagy	SseF	2	HeLa	Interacts with the small GTPase Rab1A in host cells to impair autophagy initiation	Feng et al., 2018
Autophagy	SseG	2	HeLa	Interacts with the small GTPase Rab1A in host cells to impair autophagy initiation	Feng et al., 2018
Autophagy	AvrA	1	HCT116	Suppression of autophagy by reducing Beclin-1 expression through the JNK pathway	Jiao et al., 2020
Autophagy	SrrB	2	BMDMs	Targeting Sirt1/LKB1/AMPK for lysosomal degradation, which enables sustained mTOR-activation and inhibition of autophagy	Ganesan et al., 2017
Autophagy	SifA	2	HeLa	Inhibiting retrograde trafficking of M6PR and lysosome function	McGourty et al., 2012

## 6 Crosstalk between cell death programs

Furthermore, the interplay between *Salmonella* effector proteins and the phenomenon of cross-regulation among different cell death pathways gives rise to the manifestation of hybrid forms of PCD. The interconnectedness of apoptosis and necroptosis pathways is primarily facilitated by the involvement of Caspase-8, whereby the activation of extrinsic death receptors mediated by the RIPK1/FADD/Caspase-8 complex can yield diverse signaling outcomes. In the absence of inhibition of RIPK or Caspase, the dominant mechanism of cell survival was through the activation of anti-apoptotic programs mediated by NF-κB. Suppression of RIPK resulted in the initiation of extrinsic apoptosis through Caspase-8 homodimerization. Conversely, inhibition of Caspase-8 facilitated necroptosis by promoting the oligomerization of RIPK1 and RIPK3 (Demarco et al., 2020). Necroptosis interacts with focal death signaling pathways via the effector protein MLKL. Additionally, the activation of cytoplasmic ZBP1 leads to the phosphorylation of MLKL, which in turn forms a pore in cellular membranes, including the plasma membrane. The activation of the NLRP3 inflammasome, triggered by subsequent K^+^ efflux mediated by MLKL, leads to the assembly of inflammasomes and cleavage of Caspase-1, resulting in the formation of GSDM-D pores and the release of IL-1β and IL-18 (Frank and Vince, 2019). Similarly, both apoptosis and pyroptosis involve the activation of Caspase proteases, which may have common evolutionary origins. As a result, these processes can interact at various levels. For example, the pyroptotic Caspase-1 protease can cleave the Bcl-2 family member Bid, leading to mitochondrial outer membrane permeabilization (MOMP) and subsequent activation of apoptotic signaling (Bock and Tait, 2020). Apoptosis-induced Caspase-8 has the capability to interact with ASC, resulting in Caspase-8-dependent pyroptotic activation (Fink and Cookson, 2007). Additionally, the transcription of NLRC4 and NLRP3 inflammasomes driven by apoptosis can facilitate Caspase-1 activation, leading to the hydrolysis of the pyroptotic execution protein GSDMD and subsequent formation of gasdermin pores, thereby inducing morphological characteristics associated with pyroptosis (Wemyss and Pearson, 2019). Emerging evidence indicates that autophagy and apoptosis can also engage in interactions, exhibiting antagonistic or cooperative effects, thereby exerting distinct influences on cellular destiny. Regulators involved in the interplay between autophagy and apoptosis have been identified, including the Bcl-2 protein family, Caspases, Beclin1, NF-κB, and certain microRNAs. These regulators exhibit dual coordinated roles at the transcriptional level. Additionally, kinase signaling pathways such as JNK and PI3K/Akt/mTOR have been demonstrated to play a significant role in mediating the interaction between autophagy and apoptosis (Wemyss and Pearson, 2019; Snyder and Oberst, 2021). Notably, recent research has revealed a significant finding that *Salmonella* infection has the capability to induce PANoptosis, a complex interplay involving apoptosis, pyroptosis, and necroptosis. Moreover, it has been discovered that the *Salmonella* effector SopF plays a crucial role in regulating the PANoptosis of intestinal epithelial cells, thereby exacerbating systemic infection (Christgen et al., 2020; Yuan et al., 2023). The intricate molecular network and the pleiotropic nature displayed by these regulators are expected to be influenced by the cellular milieu and diverse downstream targets. Alternatively, inhibition of specific proteins in the pathway may activate multiple PCD pathways, thereby enabling the organism to accomplish its defense objectives through alternative mechanisms.

## 7 Conclusions and future prospects

During *Salmonella* infection, bacteria manipulate PCD in immune and non-immune cells through multiple mechanisms, including interference with cell signaling pathways and regulation of cellular metabolism. The interaction between these pathways of cell death implies that, similar to numerous immune pathways, cell death signals have undergone evolutionary adaptation and diversification in response to the selective pressure imposed by *Salmonella* infection. The flexible utilization and interconnectedness of various cell death pathways serve as a defense mechanism against intracellular infection. Through extensive research on the mechanism of action of *Salmonella* and PCD, our understanding of the intricate complexity and diverse nature of *Salmonella* and cell programmed has significantly advanced. However, numerous unanswered questions persist. For instance, which PCD pathway is predominantly activated during *Salmonella* infection? How do the multiple virulence factors produced by *Salmonella* synchronize and regulate these PCD mechanisms? What are the principal regulatory effector proteins implicated in these processes? Do effector proteins possess comparable sites for bulk activation or inhibition, facilitating precise regulation of PCD processes through ubiquitination modification? Furthermore, what is the safety profile of clinical drugs targeting PCD in the treatment of *Salmonella* infections, considering the escalating issue of antibiotic resistance? In an era of increasing antibiotic resistance, it is imperative to comprehend the significance of PCD within the innate immune system, as well as to unravel the intricate molecular mechanisms employed by bacteria to evade and exploit PCD. This comprehension is of utmost importance in the regulation of bacterial infections through PCD, the identification of novel drug targets for intracellular bacterial infections, and the formulation of innovative strategies for the prevention and control of bacterial proliferation.

## Author contributions

YZ: Supervision, Writing—original draft. MX: Investigation, Writing—original draft. YG: Visualization, Writing—review & editing. LC: Writing—review & editing. WV: Supervision, Writing—review & editing. QX: Conceptualization, Writing—review & editing. LL: Formal analysis, Investigation, Writing—review & editing.
